# ﻿Two new *Drawida* (Oligochaeta, Moniligastridae) earthworms from Vietnam

**DOI:** 10.3897/zookeys.1099.72112

**Published:** 2022-05-03

**Authors:** Tung T. Nguyen, Dang H. Lam, Binh T. T. Tran, Anh D. Nguyen

**Affiliations:** 1 Department of Biology, School of Education, Can Tho University, Can Tho City, Vietnam Can Tho University Can Tho Vietnam; 2 Faculty of Biology, Hanoi University of Education, Xuan Thuy Str., Caugiay, Hanoi, Vietnam Hanoi University of Education Hanoi Vietnam; 3 Department of Soil Ecology, Institute of Ecology and Biological Resources, Vietnam Academy of Science and Technology, 18, Hoangquocviet, Caugiay, Hanoi, Vietnam Institute of Ecology and Biological Resources Hanoi Vietnam; 4 Graduate University of Science and Technology, Vietnam Academy of Science and Technology, 18, Hoangquocviet, Caugiay, Hanoi, Vietnam Graduate University of Science and Technology Hanoi Vietnam

**Keywords:** Biodiversity, bio-investigation, COI, new species, taxonomy

## Abstract

Two new earthworm species are described, namely *Drawidaangiang***sp. nov.** and *Drawidacochinchina***sp. nov.** The former can be recognized by having male pores on spiniform penises in intersegment 10/11, an erect and sac-shaped spermathecal atrium, glandular prostate, the capsule coiled one round, the vas deferens strongly coiled but small, two large, round, genital markings on segments ix–x, and three gizzards in xiii–xv. The latter species is distinguished in having the male pores placed on highly elevated, backwardly directed, conical penises in 10/11, a slender spermathecal atrium, a glandular prostate, a somewhat folded capsule, the vas deferens strongly coiled as a bunch and equal size to the testis sacs, a pair of genital markings located closely anterior to the penises with 1–3 additional ones in xi–xii, and three or four gizzards in xiii–xvi. The DNA barcode fragment of the COI gene was extracted for each species, and the COI genetic distances and phylogenetic analysis also supported two new species..

## ﻿Introduction

To date, the earthworms of Vietnam are well known with 245 species and subspecies described (Nguyen TT et al. 2016a, 2016b, 2018; Nguyen QN et al. 2020; Lam et al. 2021), of which 232 belong to the species-rich family Megascolecidae; the family Moniligastridae has been reported with only five species, although Vietnam is located in the region of origin of this family (Sims and Easton 1972). Five *Drawida* Michaelsen, 1900 species are *Drawidaannamensis* Michaelsen, 1934, *D.beddardi* (Rosa, 1890), *D.delicata* Gates, 1962, *D.chapaensis* Do & Huynh, 1993, and *D.langsonensis* Do, 1993 in Do & Huynh, 1993. Three species are only known from Vietnam: *D.annamensis*, *D.chapaensis*, and *D.langsonensis* (Nguyen TT et al. 2016b).

Most recent research on earthworms in Vietnam focuses mainly on the family Megascolecidae, especially the pheretimoid group. There are no works on the family Moniligastridae or the genus *Drawida* in Vietnam. This work, therefore, aims to contribute to a better knowledge of the genus *Drawida* through descriptions of two new species.

## ﻿Materials and methods

### ﻿Specimen collecting and preservation

Earthworms were manually searched for and collected in Vietnam for a decade during the rainy season, September to November, in 2010–2020. After collection, specimens were cleaned with tap water, killed in 2% formalin, temporally fixed in 4% formalin for 12 hours, then transferred to fresh 4% formalin for long-term preservation. Specimens for molecular study were preserved in 95% ethanol. Specimens including holotypes and paratypes were deposited in Laboratory of Zoology, Department of Biology, Can Tho University (**CTU**). Some were shared with the Department of Soil Ecology, Institute of Ecology and Biological Resources (**IEBR**), Hanoi, Vietnam.

### ﻿Morphological examination

Material was examined under a Motic Digital microscope (model: DM143-FBGG-C) and dissected from the dorsal side for internal observations. Transverse body sections were processed using the classical method of Hematoxylin & Eosin. Selected segments were cleaned and dehydrated using graded ethanol concentrations. Segments were imbedded with paraffin, then cut using a microtome Sakura Accu SRM 200CW. The cut sections were stained using Hematoxylin & Eosin Y (Feldman and Wolfe 2014) and transferred onto glass slides and mounted.

Color images were taken using a camera attached directly to the microscope. Line drawings and color images were improved and grouped into plates using Photoshop CS6.

### ﻿DNA extractions, PCR, and sequencing

Total genomic DNA was extracted from several body segments using a DNeasy Blood & Tissue Kit (Qiagen TM). A fragment of the mitochondrial gene, cytochrome c oxidase subunit I (COI), was amplified using polymerase chain reaction (PCR). Universal primers LCO-1490 and HCO-2198 (Folmer et al. 1994) were used to amplify a 680 bp fragment of the COI region. PCR conditions for amplification of the COI gene were as follows: an initial denaturation at 95 °C for 2 minutes followed by 36 cycles of 95 °C for 20 seconds, 42 °C for 45 seconds, and 72 °C for 1 minute, and a final extension at 72° for 5 minutes. Successfully amplified samples were sent for purifying and sequencing at the FirstBase Company (Malaysia). The same primers for the initial PCR were also used as sequencing primers.

Each sequence chromatogram was manually checked using BioEdit v.7.1 (Hall 1999), and the identity confirmed by a BLAST search (Zhang et al. 2000). All confirmed sequences were aligned using multiple sequence alignment with the program ClustalX v. 2.0 (Larkin et al. 2007).

After trimming, the final COI dataset consists of 580 bp from 47 samples of 19 species including the outgroup, *Pontoscolexcorethrurus* (Table 1). The nucleotide frequencies of A, T, G, and C were 25.6%, 34.2%, 18.4%, and 21.8%, respectively. The GC content was 41.5%. The dataset contained 242 (41.7%) parsimony informative and 252 (43.4%) variable sites.

**Table 1.** Species vouchers and GenBank accession numbers of species used for analyses.

Species	Locality / species voucher	Accession number	Source
*Drawidaangiang* sp. nov.	Vietnam/CTU-EW.181.018EW	ON303834	This study
*Drawidacochinchina* sp. nov.	Vietnam/ CTU-EW.032.019EW	ON303833	
*Drawidacochinchina* sp. nov.	Vietnam/ CTU-EW.032.19a	ON303831	
*Drawidacochinchina* sp. nov.	Vietnam/ CTU-EW.032.19b	ON303832	
*Drawidanepalensis* Michaelsen, 1907	Vietnam/ CTU-EW.031.06	ON303830	
*Drawidanepalensis* Michaelsen, 1907	Vietnam/ CTU-EW.031.07	ON303828	
*Drawidanepalensis* Michaelsen, 1907	Vietnam/ CTU-EW.031.08	ON303829	
*Drawidanepalensis* Michaelsen, 1907		MT472588	
*Drawidanepalensis* Michaelsen, 1907		MT570063	
*Drawidanepalensis* Michaelsen, 1907		MT570064	
*Drawidanepalensis* Michaelsen, 1907		MH845467	
*Drawidajaponica* Michaelsen, 1892		EF077597	Huang et al. (2007)
*Drawidahattamimizu* Hatai, 1930		AB543219	
*Drawidahattamimizu* Hatai, 1930		AB543220	
*Drawidahattamimizu* Hatai, 1930		AB543224	
*Drawidaghatensis* Michaelsen, 1910	India/ IEW386-17		Thakur et al. (2021)
*Drawidaghatensis* Michaelsen, 1910	India/ IEW432-17		
*Drawidaghatensis* Michaelsen, 1910	India/ IEW433-17		
*Drawidaghatensis* Michaelsen, 1910	India/ IEW434-17		
*Drawidaghatensis* Michaelsen, 1910	India/ IEW435-17		
*Drawidaghatensis* Michaelsen, 1910	India/ IEW436-17		
*Drawidabrunnea* Stephenson, 1915	India/ IEW388-17		
*Drawidaimpertusa* Stephenson, 1920	India/ IEW391-17		
*Drawidaimpertusa* Stephenson, 1920	India/ IEW393-17		
*Drawidaimpertusa* Stephenson, 1920	India/ IEW447-17		
*Drawidaimpertusa* Stephenson, 1920	India/ IEW448-17		
*Drawidaimpertusa* Stephenson, 1920	India/ IEW424-17		
*Drawidacircumpapillata* Aiyer, 1929	India/ IEW420-17		
*Drawidatravancorense* Michaelsen, 1910	India/ IEW425-17		
*Drawidarobusta* (Bourne, 1887)	India/ IEW444-17		
*Drawidarobusta* (Bourne, 1887)	India/ IEW445-17		
*Drawidascandens* Rao, 1921	India/ IEW451-17		
*Drawidanilamburensis* (Bourne, 1887)	India/ IEW459-17		
*Drawidagracilis* Gates, 1925		JN793516	
*Drawidagracilis* Gates, 1925		JN887887	
*Drawidabullata* Gates, 1933		JN793527	
*Drawidabullata* Gates, 1933		JN887894	
*Drawidagistigisti* Michaelsen, 1931		JQ405262	
*Drawidaghilarovi* Gates, 1969		KY711477	Ganin and Atopkin (2018)
*Drawidaghilarovi* Gates, 1969		KY711499	
*Drawidaghilarovi* Gates, 1969		KY711501	
*Drawidaghilarovi* Gates, 1969		KY711517	
*Drawidakoreana* Kobayashi 1936		KR047039	Shen et al. (2015)
*Drawidakoreana* Kobayashi 1936		MH845538	Yuan et al. (2019)
*Drawidakoreana* Kobayashi 1936		MH882566	
*Drawidakoreana* Kobayashi 1936		MH882855	
*Pontoscolexcorethrurus* (Müller, 1856)		JN260736	

The K2P (Kimura 2 parameters) genetic distance was calculated in MEGA 7.0 (Kumar et al. 2016. The phylogenetic tree was reconstructed using a maximum-likelihood analysis with the best model chosen using ModelFinder (Kalyaanamoorthy et al. 2017) performed in IQTREE v.1.6.2 for Windows (Nguyen LT et al. 2015). The best model was GTR + F + I + G4 with BIC score = 12628.239 and –lnL = 6005.512.

### ﻿Abbreviations

**ag** accessory gland;

**amp** ampulla;

**atr** atrium;

**cl** clitellum;

**CTU** Can Tho University;

**gm** genital markings;

**mp** male pore;

**os** ovi sac;

**pc** penial chamber;

**pn** penis;

**prg** prostate gland;

**sp** spermathecal pore;

**ts** testis sac;

**vd** vas deferens.

## ﻿Results

### ﻿Molecular analysis

The genetic distance between new species and other *Drawida* ranges from 21.5% (*D.cochinchina* and *D.japonica*) to 29.3% (*D.angiang* and *D.scandens*).

The interspecific divergence among *Drawida* species ranges from 16.3% (*D.impertusa* and *D.robusta*) to 31.1% (*D.nilamburensis* and *D.japonica*). The average interspecific distance in the genus *Drawida* was previously reported as 22%, and the maximum one was 34.3% between *D.impertusa* and *D.deshayesi* (Thakur et al. 2021). The *p*-genetic distance was also known to range from 18% between *D.koreana* and *D.japonicajaponica* to 24.82% between *D.koreana* and *D.gracilis* (Zhang et al. 2020).

**Figure 1.**
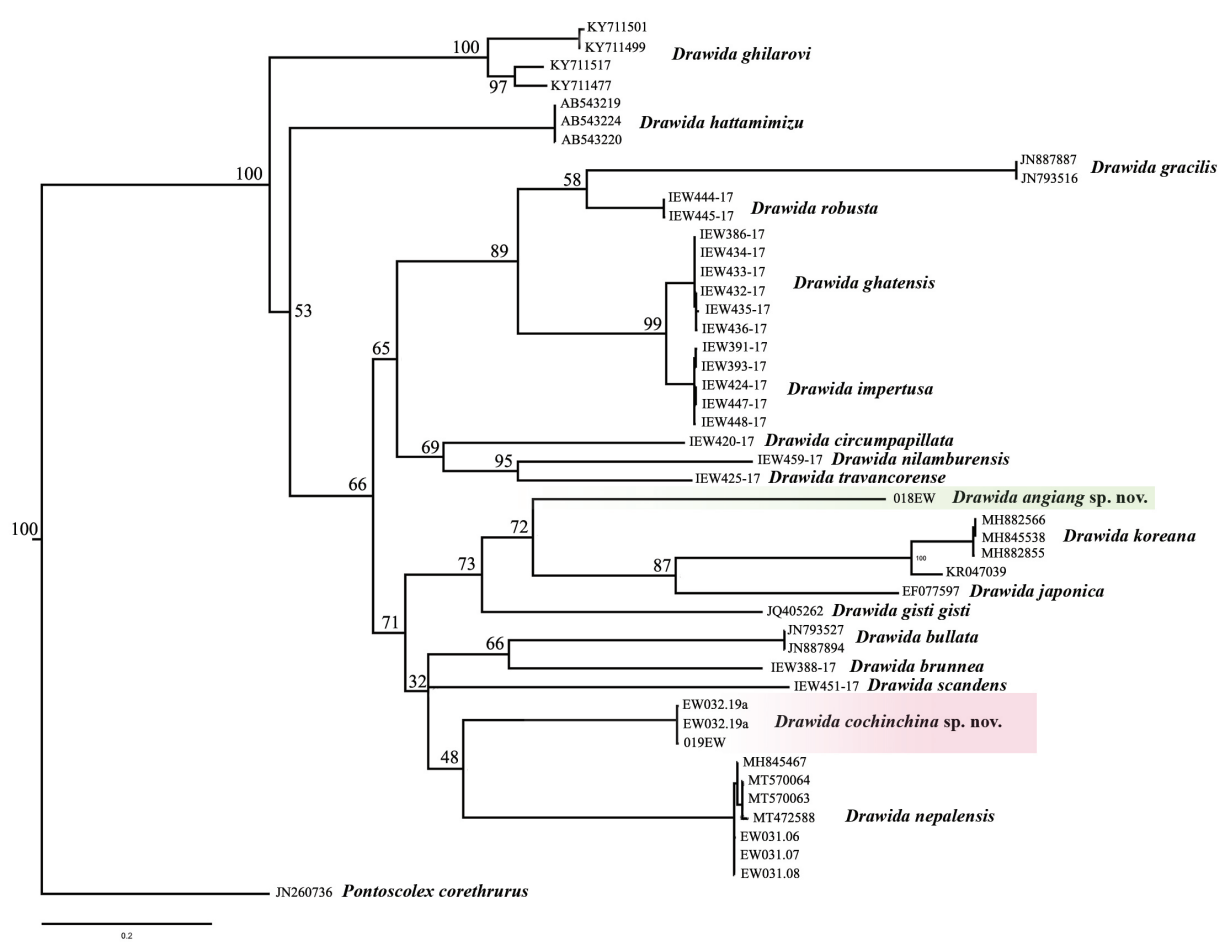
Phylogenetic diagram inferred from 580 bp COI dataset using maximum likelihood analysis. Numbers at node show the bootstrap values.

In the maximum-likelihood tree (Fig. 1), *Drawidaangiang* sp. nov. is closely related to both East Asian species, *D.japonica* and *D.koreana*. The relationship is moderately supported with a bootstrap value of 72%. On the contrary, *D.cochinchina* sp. nov. is clustered as a sister species to the South Asian *D.nepalensis*, but the relationship is poorly supported by bootstrap and Bayesian values (48%). In other words, two species, *D.cochinchina* and *D.nepalensis* are distantly related; there must be intermediate species, which are still unknown and need to be discovered. The presence of *D.cochinchina* in Vietnam also indicates that the genus has a long presence in Asia, with some dispersal within Asia.

### ﻿Taxonomic part

#### Family Moniligastridae Claus, 1880

##### ﻿Genus Drawida

Taxon classificationAnimaliaMoniligastridaMoniligastridae

Michaelsen, 1900

897693BA-F982-5035-8CBE-720729CCCE9A

Drawida Michaelsen, 1900: 114; Stephenson 1930: 814; Gates 1972: 244.

###### Type species.

*Moniligasterbarwelli* Beddard, 1886.

###### Distribution.

India, Myanmar, Malay Peninsula, Thailand, Cambodia, Laos, Vietnam, China, Korea, Japan, Philippines, Malaysia, Indonesia, Borneo, Sumatra, Java, Sri Lanka, Taiwan, Far East of Russia, Caribbean, Australia, and various Pacific islands (Gates 1972; Blakemore 2002; Chang et al. 2009; Zhang et al. 2020).

##### ﻿ Drawida angiang

Taxon classificationAnimaliaMoniligastridaMoniligastridae

sp. nov.

D4BF856B-5094-51C0-A126-0FFA506FA8C8

http://zoobank.org/CBBB4135-C65E-459C-A874-93CF63728135

Fig. 2

Drawida sp. 1. — Nguyen TT 2013: 100; 2014: 113.

###### Material examined.

***Holotype*.** Vietnam • clitellate; An Giang Province, Tinh Bien District, Nhon Mountain; 10.5882°N, 104.9506°E; 56 m a.s.l.; 07 Nov. 2010; Nguyen Thanh Tung leg.; natural forest; CTU-EW.181.h01.

***Paratypes*.** Vietnam • 2 clitellates, 12 aclitellates; An Giang Province, Tinh Bien District, Nhon Mountain; 10.5882°N, 104.9506°E; 56 m a.s.l.; 07 Nov. 2010, coll. Nguyen Thanh Tung leg; natural forest; CTU-EW.181.p02.

###### Other material.

Vietnam • 8 juveniles; An Giang Province, Tinh Bien District, Tinh Bien town; 10.5895°N, 104.9501°E; 24 m a.s.l.; 19 Oct. 2020; Nguyen Thanh Tung leg.; near a pond inside a *Citrusgrandis* garden; CTU-EW.181.03.

###### Diagnosis.

Body cylindrical, small-medium size, length 72–116 mm, diameter 3.6–4.0 mm, 170–221 segments. Setal formula aa: ab: bc: cd: dd = 6.5–7.0: 1: 6.5–7.0: 1: 35–37. No dorsal pores. Clitellum within ix–xiv. Male pores located on tip of spiniform penis in 10/11. Spermathecal pores located median to c. Genital markings, present, two, circular, on ix and x. Spermathecal atrium erect in vii, sac-like. Testis sacs in 10, much larger than the coils of vas deferens. Prostate glandular, glandularity reduced; prostatic capsule cylindrical, somewhat folded. Gizzards 3, in xiii–xv.

###### Description.

**External**: body cylindrical, gradually tapering towards tail, small–medium-sized, length 72–116 mm, diameter 3.6–4.0 mm, 170–221 segments (holotype: length 80 mm, diameter 3.9 mm, 199 segments).

**Coloration**: body general darkish grey on both dorsum and ventrum, but greener toward telson. Setae lumbricine, with eight setae more concentrated on ventrum; setal formula: aa: ab: bc: cd: dd = 6.5–7.0: 1: 6.5–7.0: 1: 35–37. Prostomium prolobous. Dorsal pores absent. Clitellum annular, within ix–xiv, reddish brown. Spermathecal pores located in intersegmental furrow 7/8, median to c. Female pore hardly visible, paired in intersegment 11/12. Male pores located in intersegment 10/11, between setae b and c, somewhat spiniform penis exposed or not. Genital marking present, two, large circular markings, highly elevated from body surface, located on setal line b on segment ix and medio-ventral segment x, respectively. Nephridiopores anterior margin of segments iv onwards, in d lines, especially clear from vi to xii.

**Internal**: no pigmentation. Septa 4/5/6/7/8/9/10 thick, 10/11 thin. Gizzards 3, in xiii–xv. Nephridia holoic, from segment iv onwards. Intestinal origin at xvii; intestinal caeca absent. Last hearts in ix. Typhlosole absent. Spermathecae paired in viii, spermathecal ampulla oval, without diverticulum; spermathecal ducts strongly twisted and coiled, going through the septum 7/8 and joinomg atrium in vii subentally; spermathecal atrium erect, sac-like. No accessory glands in spermathecal region. Prostate glandular, glandularity strongly reduced; prostatic capsule cylindrical, somewhat folded. Testis sacs, paired, large, located on posterior side of septum 9/10, much larger than coils of vas deferens; vas deferens twisted and strongly coiled, ending at ental ends of prostate capsule which basally connect to penial pouch. Ovaries on septum 10/11; ovisacs sac-shaped in xiii and xiv. Accessory glands in ix and x in correspondence with genital markings outside.

###### DNA characters.

The COI fragment was uploaded to GenBank with an accession number ON303834. The new species has a close COI identity of 81.5% with *D.koreana* (KR047039)

###### Distribution.

The species was previously recorded from Kien Giang Province (Da Dung Mountain), An Giang Province (Ba Doi Mountain, Cam Mountain, Nhon Mountain, Phu Tan, Cho Moi), Vinh Long Province (Vung Liem), Dong Thap Province (Lai Vung, Long Thuan Island, Tan Long Island), Can Tho, Hau Giang (Phung Hiep) (Nguyen T.T. 2014 as *Dr.*sp. 1).

###### Etymology.

A noun in apposition, *angiang*, is used to emphasize the province where type specimens were collected.

###### Remarks.

The species is very similar to *D.angchiniana* Chen, 1933 from northern China (Anhwei and Kiangsu) and South Korea (Jeju Island) (Michaelsen 1931; Chen 1933; Kobayashi 1937) by the presence of genital markings in the male region and absence in the spermathecal region. However, the two species can be distinguished by the location of genital markings (ix and x vs x and xi), number of segments (170–221 vs 134–145), location of clitellum (x–xiv vs ix–xiv), and shape of the spermathecal atrium (long, enlarged distally vs short or cylindrical) (Table 2).

**Table 2.** Character comparison between *Drawidaangiang* sp. nov., *D.cochinchina* sp. nov., *D.longatria* Gates, 1925, *D.ofunatoensis* (Ohfuchi, 1938), and *D.angchiniana* Chen, 1933.

Species	*D.angiang*	*D.cochinchina*	*D.longatria ^1^*	*D.ofunatoensis ^2^*	*D.angchiniana ^3^*	*D.nepalensis ^4^*	*D.koreana ^5^*	*D.japonica ^6^*
Length (mm)	72–116	84–123	153	228–283	62–80	129–180	63–100	28
Diameter (mm)	3.6–4.0	3.3–4.9	6	≤6.5	3–5	4–5	3–4	3
Segments	170–221	101–294	208	189–242	134–145	78–130	80–90	95
Clitellum	ix–xiv	ix–xiv	x–xiii	x–xiii	x–xiii	ix–xiv	x–xiii	ix–xiii or xiv
Genital markings in	two, in ix and x	many, vary in viii, 10/11, x–xiii	paired, viii, 10/11, xii	many, vary in vii–xii	two, in x and xi	paired, vii, x, 10/11, xi	unpaired, vii–x	unpaired, vii–xiii
Gizzards	3, within xiii–xv	3 or 4 within xiii–xvi	4 within xv–xviii	4 within xii–xvii	3, sometimes 2	2–4, within xii–xx	2 or 3, xii–xiv	2, xii and xiii
Spermathecal pores	in c-line	median to c	in c-line or median to c	median to c	median to c	median to c	in c-line or median to c	In c-line or median to c
Spermathecal atrium	vii	vii	viii	absent	vii	vii	vii,	Vii
Shape of Spermathecal atrium	erect, sac-like	slender and strongly coiled as a bunch	Slender and strongly coiled as a bunch	n/a	short, cylindrical	song, sac-like	short, sac-like	small
Testis sacs	9/10	10/11	9/10	10/11	10/11	n/a	9/10	9/10
Vas deferens	strongly coiled as a bunch	strongly coiled as a bunch	strongly coiled as a bunch	coiled and twisted, but not a bunch	coiled and twisted, but not a bunch	strongly coiled as a bunch	Loosely twisted, small	coiled and twisted, but not a bunch
Prostate	glandular, but strongly reduced; cylindrical, somewhat folded	glandular, but strongly reduced; cylindrical, somewhat folded	glandular?; coiled or curve, digitiform	glandular?; roundish-shaped	muscular; cylindrical, slender	glandular, club-shaped, slender	glandular?; thumb-shaped	glandular; club-shaped and erect
Ovisacs	xii–xiii	xii–xv, sometimes xviii	n/a	xii	from xii	n/a	xii–xviii, seldom xxii or xxiii	xii–xvi
Accessory glands	ix and x	not visible	present	present	present	present	present	present

Data extracted from: ^1^Gates (1925), ^2^Blakemore et al. (2014), ^3^Chen (1933), ^5^Kobayashi (1938), ^6^Michaelsen (1892), and Blakemore and Kupriyanova (2010)

The new species, *D.japonica* (Michaelsen, 1892) and *D.koreana* Kobayashi, 1938 share several common characters, such as the presence of genital markings and accessory glands. However, the new species differs from those two species in having three gizzards from xiii, the spermathecal atrium erect, the vas deferens strongly coiled as a bunch, and the prostate cylindrical and somewhat folded, while *D.japonica* and *D.koreana* have two to three gizzards from xii, the spermathecal atrium short and small, the vas deferens not into a bunch (coiled or loosely twisted), and the club-shaped or thumb-shaped prostate (Table 2).

Compared to other *Drawida* species recorded in Vietnam, *D.annamensis*, *D.chapaensis*, *D.delicata*, *D.langsonensis*, and *D.beddardi*, the new species is clearly distinguished by having genital markings in ix and x, and the spermathecal atrium and spermathecal ducts strongly twisted and coiled, while all other species have no genital markings, and the spermathecal atrium and spermathecal ducts simply undulated.

**Figure 2.**
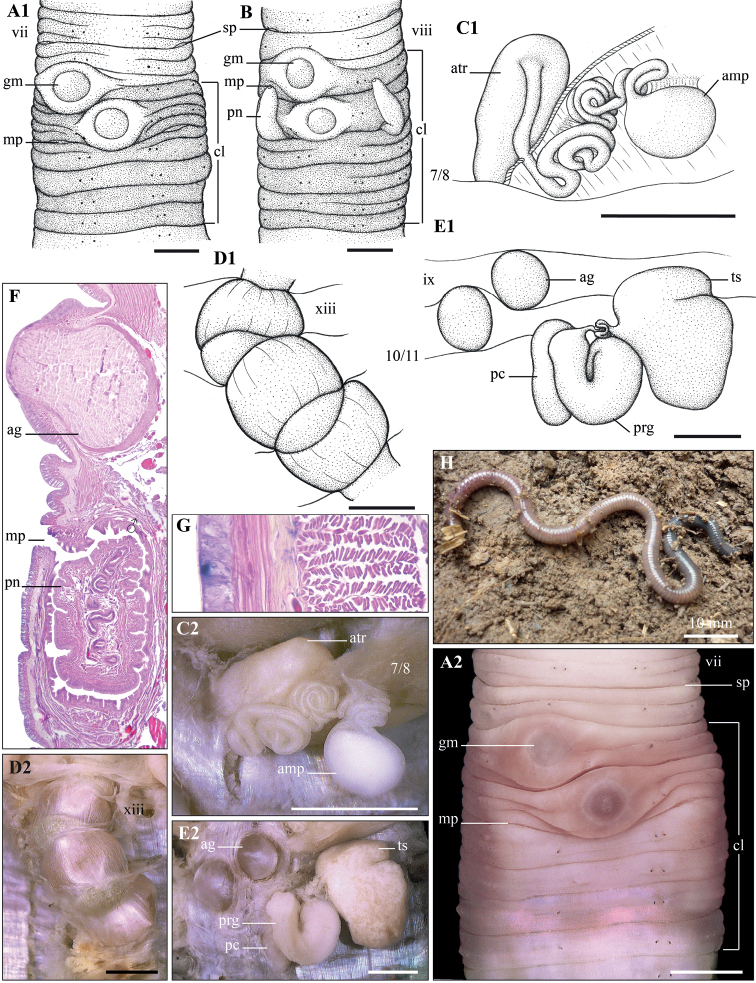
*Drawidaangiang* sp. nov. Holotype (CTU-EW.181.h01) **A1, A2** clitellum region, ventral view **B** clitellum region, with penis **C1, C2** right spermatheca **D1, D2** oesophagous gizzards **E1, E2** right male sexual system **F** longitudinal section of clitellum region **G** transverse section of body wall **H** living specimen. Scale bars: 1 mm.

##### ﻿ Drawida cochinchina

Taxon classificationAnimaliaMoniligastridaMoniligastridae

sp. nov.

33E5A121-7E22-5397-80CF-70A9E8890B17

http://zoobank.org/02322457-3AC4-4E1A-BABC-1994958EE562

Fig. 3

Drawida sp. 2. – Nguyen TT 2013: 100; 2014: 113.

###### Material examined.

***Holotype*.** Vietnam • clitellate; Dong Nai Province, Xuan Loc District, Xuan Hoa Commune; 10.7931°N, 107.5257°E; 88 m a.s.l.; 12 Sep. 2012; Nguyen Van Thang leg. (long-term tree plantation CTU-EW.032.h01),

***Paratypes*.** Vietnam • 8 clitellates; same data as for the holotype; CTU-EW.032.p02 • 3 clitellates; same data as for the holotype; CTU-EW.DNA.032.p02 • 8 clitellates; Tay Ninh Province, Ba Den Mountain; 11.3901°N, 106.1553°E; 149 m a.s.l.; 26 Sep. 2019, coll. Nguyen Quoc Nam leg.; *Mangifera* plantations; CTU-EW.032.p03.

###### Other material.

Vietnam • 50 clitellates; same data as for the holotype; CTU-EW.032.04 • 5 clitellates; same data as for the sample CTU-EW.032.p03; CTU-EW.032.09 • 2 clitellates, 8 aclitellates; An Giang Province, Tinh Bien District, Nhon Mountain; 10.5882°N, 104.9506°E; 56 m a.s.l.; 07 Nov. 2010; Nguyen Thanh Tung leg.; *Mangifera* plantations; CTU-EW.032.05 • 2 clitellates; An Giang Province, Tinh Bien District, Tinh Bien town; 10.5895°N, 104.9501°E; 24 m a.s.l.; 19 Oct. 2020; Nguyen Thanh Tung leg.; orange garden; CTU-EW.032.19 • 3 clitellates; same data as for the sample CTU-EW.032.19; IEBR-EW.032.19 • 2 clitellates, 17 aclitellates; Kien Giang Province, Kien Hai District, Hon Tre Island; 9.9538°N, 104.8359°E; 187 m a.s.l.; 13 Nov. 2013; Trinh Thi Kim Binh leg.; *Acacia* plantation; CTU-EW.032.18 • 3 clitellates, 10 aclitellates; Tay Ninh Province, Ba Den Mountain; 11.3944°N, 106.1499°E; 46 m a.s.l.; Oct. 2012, Nguyen Thi Anh Ngoc leg.; *Mangifera* plantations; CTU-EW.032.06 • 5 clitellates; Ho Chi Minh City, Hoc Mon District, Tan Hiep Commune; 10.9142°N, 106.5662°E; 2 m a.s.l.; 24 Sep. 2019; Nguyen Quoc Nam leg.; bushes; CTU-EW.032.07 • 1 clitellate; Tay Ninh Province, Tan Chau District, Tan Hiep Commune; 11.6024°N, 106.1144°E; 44 m a.s.l.; 25 Sep. 2019; Nguyen Quoc Nam leg.; rubber plantation; IEBR-EW. 032.10 • 35 clitellates; Tay Ninh Province, Trang Bang District, Loc Hung Commune; 11.0775°N, 106.4000°E; 24 Sep. 2019; Nguyen Quoc Nam leg.; rice field; CTU-EW.032.08 • 3 clitellates; same data as for the sample CTU-EW.032.08; IEBR-EW.032.08 • 2 matures, 19 aclitellates; Ba Ria – Vung Tau Province, Con Son Island, 8.7008°N, 106.6175°E; 10 m a.s.l.; 19 Nov. 2019; Nguyen Thanh Tung & Nguyen Thi Bao Ngoc leg.; bushes; CTU-EW.032.11 • 3 clitellates; same data as for the sample CTU-EW.032.11; IEBR-EW.032.11 • 14 clitellates; Ba Ria – Vung Tau Province, Dinh Mountain; 10.51111 N, 107.12694 E; 27 Oct. 2016; Nguyen Quoc Nam leg.; natural forest; CTU-EW.032.13 • 3 clitellates; Dong Nai Province, Cam My District, Lam Son Commune; 10.83944 N, 107.26508 E; 16 Oct. 2019; Nguyen Quoc Nam leg.; rubber plantation; IEBR-EW.032.12 • 12 clitellates, 18 aclitellates; Binh Duong Province, Dau Tieng District, Dinh An Commune; 11.3765°N, 106.4234°E; 27 Oct. 2017; Nguyen Quoc Nam leg.; rubber plantation; CTU-EW.032.14 • 2 clitellates, 19 aclitellates; Binh Duong Province, Dau Tieng District, Minh Thanh Commune; 11.3811°N, 106.5159°E; 37 m a.s.l.; 27 Oct. 2017; Nguyen Quoc Nam leg.; cashew plantation; CTU-EW.032.15. CTU-EW.032.16 • 12 clitellates; Dong Nai Province, Long Thanh District, Long Phuoc Commune; 10.7018°N, 107.0040°E; 10 m a.s.l.; 12 Oct. 2012; Le Van Nhan leg.; long-term tree plantation; CTU-EW.032.17.

###### Diagnosis.

Body cylindrical, small-medium in size, length 84–123 mm, diameter 3.3–4.9 mm, 101–294 segments. Setal formula aa: ab: bc: cd: dd = 6.2–7.0: 1: 7.0–8.5: 1: 33–35. A pair of spermathecal pores in ventro-lateral intersegment 7/8, close to seta c. Genital markings, variable, one or two pairs, in viii and ix, located between seta b and c, (sometimes with additional one or two genital markings in medio-ventral viii and ix), one pair closely anterior to penises, and additional 1–3 ones in xi–xii. Male pores located on the top of highly elevated, posteriorly directed, conical penises in 10/11. Spermathecal atrium tubular, strongly coiled in vii. Testis sacs in x, large, in equal size to the coils of vas deferens. Prostate glandular, glandularity reduced; prostatic capsule cylindrical-shaped, somewhat folded. Gizzards 3–4, in xiii–xvi.

###### Description.

**External**: body cylindrical, small-medium size, length 84–123 mm, diameter 3.3–4.9 mm, 101–294 segments (holotype: length 95, diameter 5.1, 198 segments).

**Coloration**: body light grey, uniformly color in both ventrum and dorsum. Prostomium undeveloped. No dorsal pores. Setae lumbricine, with eight setae distributed round body, setal formula aa: ab: bc: cd: dd = 6.2–7.0: 1: 7.0–8.5: 1: 33–35. Clitellum annular, within ix–xiv, reddish brown. Spermathecal pores paired, in ventro-lateral intersegmental furrow 7/8, close to seta c. Genital markings present, variable, one or two pairs in viii and ix, located between setae b and c, sometimes with an additional one or two in medio-ventral viii and ix, one pair anterior to penis in x, and additional 1–3 in xi–xii. Female pore hardly visible. Male pores located in intersegmental furrow 10/11, between setae b and c, closed to seta c, on the top of highly elevated, backwardly directed, conical penises in 10/11. Nephridiopores anterior margin of segments iv onwards, in d lines, especially clear from vii to xv.

Internal: Septa 5/6/7/8/9 thick, 9/10 and subsequence septa thin. Gizzards three or four in xiii–xvi. Last hearts in ix. Intestinal origin at xvi or xvii. Spermathecae paired, on viii, spermathecal ampulla oval; spermathecal ducts coiled and twisted, passing through septum 7/8, and ending at ectal end of atrium; Spermathecal atrium tubular, strongly coiled as a bunch in vii, mass larger than spermathecal ampulla. Prostate glandular, glandularity strongly reduced; prostatic capsule cylindrical, somewhat folded. Testis sacs paired, in x, large, sac-shaped; vas deferens strongly coiled as a bunch, equal in size to testis sacs, and entering testis sac at its ental end. Ovarian chamber complete, ovisacs well developed, in xii–xviii. Accessory glands present, but invisible.

###### DNA character.

The COI fragment was uploaded to GenBank with accession numbers ON303831, ON303832, ON303833. The new species has a close COI identity of 81% with *D.ghilarovi* (KY711506)

###### Distribution.

The species was also found in Kien Giang (Da Do, Da Dung, and Ta Bang Mountains), An Giang (Tinh Bien District), Vinh Long (Vung Liem District), Ho Chi Minh City (Hoc Mon, Binh Chanh, and Cu Chi Districts), Tay Ninh (Ba Den Mountain, Trang Bang District), Binh Duong (Dau Tieng and Bau Bang Districts), Dong Nai (Xuan Loc, Long Thanh, and Cam My Districts), Ba Ria Vung Tau (Dat Do Districts, Ba Ria City, and Con Son Island) (Nguyen T.T. 2014 as *Dr.*sp. 2).

###### Etymology.

The noun *cochinchina* (= southern Vietnam) is used in apposition is to accentuate its wide distribution in southern Vietnam.

###### Remarks.

The new species is very similar to *D.longatria* Gates, 1925 in having genital markings in 10/11, the presence of a spermathecal atrium, and the spermathecal ducts being twisted and strongly coiled. However, it differs from *D.longatria* in having prostate capsule cylindrical, somewhat folded, three or four esophageal gizzards in xiii–xvi, the spermathecal atrium in vii, testis sacs in 10/11, ovisacs well developed in xii-xvii, and having hidden accessory glands. On the contrary, *D.longatria* has the prostate capsule digitiform, four esophageal gizzards in xv–xviii, the spermathecal atrium in viii, testis sacs in 9/10, ovisacs in xi–xiv, and obvious accessory glands.

The new species is also similar to *D.ofunatoensis* (Ofuchi, 1938) in having paired genital markings and testis sacs in septum 10/11. However, it differs in having the clitellum within ix–xi, the spermathecal atrium and seminal ducts twisted and strongly coiled, and the prostate cylindrical and strongly folded. *Drawidaofunatoensis* has the clitellum located in x–xiii, the spermatheca lacking an atrium, and the male atrium globular.

The new species is somewhat similar to *D.nepalensis* in having the clitellum within ix–xiv, the presence of genital markings, spermathecal pores located median to c, and the vas deferens strongly coiled as a bunch. However, it differs from *D.nepalensis* in having the spermathecal atrium slender and strongly coiled as a bunch, one gizzard per segment (three to four within xiii–xvi), a folded cylindrical prostate, and hidden accessory glands. On the contrary, *D.nepalensis* has each gizzard pass through several segments (two to four within xii–xx), the spermathecal atrium stouter and sac-like, the prostate club-shaped, and obvious accessory glands.

Compared to the other five *Drawida* species recorded in Vietnam, *D.annamensis*, *D.chapaensis*, *D.delicata*, *D.langsonensis*, and *D.beddardi*, this new species is clearly distinguished by having paired genital markings and the spermathecal atrium and spermathecal ducts strongly twisted and coiled, while all other species have no genital markings and the spermathecal atrium and spermathecal ducts are simply undulated.

**Figure 3.**
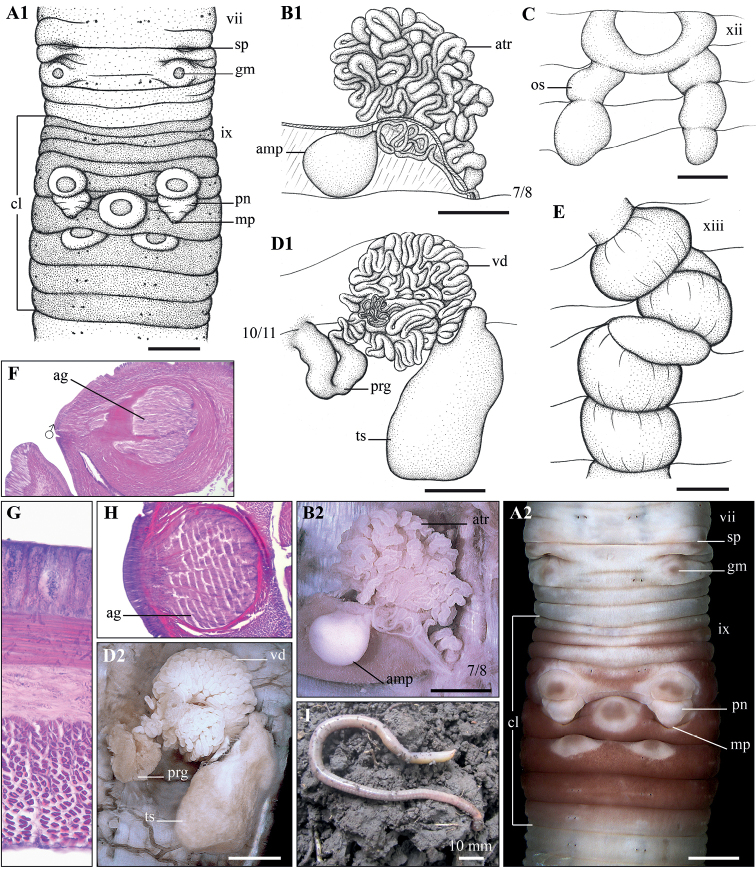
*Drawidacochinchina* sp. nov. Holotype (CTU-EW.032.h01) **A1, A2** clitellum region, ventral view **B1, B2** left spermatheca **C** ovisacs **D1, D2** right male sexual system **E** oesophagous gizzards **F** longitudinal section of clitellum region **G** transverse section of body wall **H** transverse section of genital marking **I** living specimen. Scale bars: 1 mm.

## ﻿Conclusions

The discovery of two new species of *Drawida* brings the number of species in Vietnam to seven. However, due to the placement of Vietnam in region of origin of the genus *Drawida*, this number of species does not reflect the true biodiversity in this country. It is, therefore, suggested that additional intensive surveys are needed to reveal more new species awaiting discovery.

## Supplementary Material

XML Treatment for Drawida

XML Treatment for Drawida angiang

XML Treatment for Drawida cochinchina

## Supplementary materials

Supplementary material 1 COI dataset Data type fas file Explanation note COI dataset. https://binary.pensoft.net/file/679739This dataset is made available under the Open Database License (http://opendatacommons.org/licenses/odbl/1.0/). The Open Database License (ODbL) is a license agreement intended to allow users to freely share, modify, and use this Dataset while maintaining this same freedom for others, provided that the original source and author(s) are credited.Tung T. Nguyen, Dang H. Lam, Binh T.T. Tran, Anh D. Nguyen
